# Emotionally charged events as a trigger for the acute development of psychotic symptomatology. A case report

**DOI:** 10.1192/j.eurpsy.2024.1434

**Published:** 2024-08-27

**Authors:** P. Del Sol Calderon, A. Izquierdo de la Puente, R. Fernández, M. García Moreno, C. Delgado

**Affiliations:** ^1^Psychiatry, Hospital Puerta de Hierro; ^2^Psychiatry, Hospital Universitario Infanta Cristina, Madrid, Spain

## Abstract

**Introduction:**

A 21-year-old male presented to the emergency room due to strange behavior

**Objectives:**

Show how emotionally intense events can be a stress factor leading to dissociative or psychotic symptoms.

**Methods:**

Case report and literature review

**Results:**

The patient is in Spain after having attended the meeting with the Pope at the World Youth Day in Portugal. He is an engineering student who, in the week prior to the trip, had high levels of stress related to exam time. He also explained that he had recently had conflicts with his partner. In the psychocopathological examination of the patient, a global insomnia of 3 days of duration stands out. In addition, a disorganized and disjointed speech focused on high concern that something bad could happen to his family and partner. In the interview he appears restless, nervous, with a perplexed contact. The patient’s companion says that he has been very worried and obsessed about his relationship with his partner, with constant doubts about asking her to marry him. It is decided to start olanzapine, receiving up to 15 mg per day. In the following interviews he shows better contact and a more organized speech.

**Conclusions:**

It is known that emotionally intense situations can be a trigger for the development of psychotic symptoms. There are different manifestations of these stressful situations such as physical symptoms like fainting or but mental symptoms are also described such as dissociative amnesias, or less frequent as in this case psychotic symptoms. They are usually of sudden onset and early remission with good response to anxiolytics or antipsychotics.

**Disclosure of Interest:**

None Declared

